# No evidence lithium supplementation extends lifespan in male *Drosophila melanogaster*

**DOI:** 10.1007/s10522-026-10412-5

**Published:** 2026-03-10

**Authors:** Andrew William McCracken, Joe Garden, Nicola White, Stuart Wigby

**Affiliations:** Institute of Infection, Veterinary & Ecological Sciences, Faculty of Health and Life Sciences, University of Liverpool, Liverpool, UK

**Keywords:** Lithium, Drosophila, Lifespan, Reproduction, Male, Geroprotector

## Abstract

Pharmacological modulation of ageing is viewed as a viable route to extending lifespan and healthspan, yet the efficacy of putative geroprotectors may depend strongly on physiological and environmental context. Lithium chloride (LiCl) has been reported to extend lifespan in several model organisms, but evidence remains inconsistent and the role of reproductive investment—an energetically costly and often lifespan-correlated process—has rarely been examined. We tested the effects of dietary LiCl on survival and reproductive performance in male *Drosophila melanogaster*, comparing unmated (UM) and frequently-mated (FM) males. Contrary to previous work, both a concentration previously reported as beneficial (25 mM), and a lower concentration (10 mM) reduced survival, with effects driven exclusively by UM males. FM males were comparatively protected, revealing a strong mating-dependent interaction. LiCl had no detectable effect on male late-life mating latency, sterility, or paternity share, but reduced some post-copulatory performance in FM males by increasing female remating, indicating that somatic and reproductive responses to LiCl can be decoupled. These results demonstrate that lithium’s effects on survival are strongly dependent on reproductive environment, highlighting the importance of reproductive context when evaluating its impact on lifespan.

**Supplementary Information:**

The online version contains supplementary material available at 10.1007/s10522-026-10412-5.

## Introduction

Ageing is a universal biological process that leads to the progressive decline of physiological function and increased vulnerability to disease and mortality (Lopez-Otin et al. 2013). As global populations continue to age, identifying interventions that can extend lifespan and healthspan has become a central goal of biomedical research (Gonzalez-Freire et al. 2020). A growing number of compounds, often termed geroprotectors, have been proposed to slow the ageing process by targeting conserved molecular pathways involved in cellular maintenance and stress resistance. Importantly, interventions like metformin and rapamycin, targeting evolutionarily conserved pathways have shown promise in multiple organisms (Bjedov et al. 2010; Cabreiro et al. 2013; Eisenberg et al. 2009; Harrison et al. 2009; Martin-Montalvo et al. 2013), supporting the concept that pharmacological modulation of ageing is feasible.

Among these, lithium chloride (LiCl) has emerged as an intriguing candidate. Long used clinically as a mood stabiliser (Cade 1949), lithium has been shown to influence key signalling pathways implicated in ageing, including glycogen synthase kinase-3 (GSK-3) inhibition (Chatterjee and Beaulieu 2022), induction of autophagy (Sarkar et al. 2005), and improved mitochondrial function (Maurer et al. 2009). Studies in model organisms have reported lifespan and healthspan benefits associated with lithium supplementation (Maurer et al. 2009; Zarse et al. 2011). In *Drosophila melanogaster*, dietary LiCl has been found to extend lifespan (Castillo-Quan et al. 2016), and in females, it may also increase fecundity (Jans et al. 2024b). Some human epidemiological data suggest that chronic lithium exposure may be associated with reduced all-cause mortality, though causal inference remains limited (Araldi et al. 2023; Fajardo et al. 2018). Note, Araldi et al., has subsequently been retracted on ethical—not scientific—grounds.

However, the geroprotective effects of lithium are not universally observed. Chronic exposure in female *Drosophila* abolishes the typical lifespan advantage (Zhu et al. 2015), and a study in mice reported insignificant effects on lifespan (Nespital et al. 2021). These discrepancies suggest that the impact of lithium may depend on interacting biological or environmental variables. Environmental and physiological factors can profoundly influence outcomes of interventions. Recent work, for example, has shown that dietary composition can modulate lithium’s efficacy (Jans et al. 2024a).

One key variable—reproductive activity—has received little attention despite imposing substantial energetic costs (Dewsbury 1982; Perry and Tse 2013), and with strong available evidence supporting the idea that reproductive activity itself can influence lifespan (Partridge and Farquhar 1981). We reasoned that the effects of lithium might therefore be contingent upon degree of reproductive investment.

Separately, while LiCl supplementation has been tested in male *Drosophila melanogaster* for its effects on longevity, its influence on reproductive success has not been examined. Because reproduction is a central component of biological fitness, assessing this trait alongside survival can reveal potential trade-offs or co-benefits that are obscured when lifespan alone is measured.

Here, we address this problem by investigating the effects of dietary LiCl supplementation on both lifespan and a comprehensive range of reproductive measures in male *Drosophila melanogaster*, comparing outcomes between frequently-mated (FM) and unmated (UM) males. Based on previous literature, we predicted that dietary LiCl supplementation may (1) extend male lifespan (Castillo-Quan et al. 2016); (2) improve late-life reproductive performance, since lifespan-extending interventions can improve late-life reproduction (Sepil et al. 2020; Dou et al. 2017). We focused on late-life reproductive performance, as key aspects of male reproductive ageing in Drosophila—including declines in sperm and seminal fluid function—are most strongly expressed at advanced ages (~ 5 weeks), particularly under sustained mating (Sepil et al. 2020). Notably, we have previously found male fertility does not collapse between early and late adulthood, indicating that late-life assays retain meaningful variation for detecting effects of treatment (McCracken et al., preprint). Moreover, diet-mediated effects on male reproductive performance are often weak or absent at young ages and only become apparent later in life, with males at 1 week old showing limited responsiveness to dietary manipulation (McCracken et al., preprint). By integrating measures of survival and late-life reproductive output, this study aimed to provide a more comprehensive assessment of lithium’s potential as a geroprotective compound.

## Materials & methods

### Fly husbandry

Focal males used were wild-type Dahomey (*Dah*) (Partridge 1980), an outbred stock originally acquired in 1970 from Benin. Scarlet (*st*^*1*^) (Tearle et al. 1989) recessive eye mutant females were used for focal mating experiments; males were additionally used for remating experiments to allow the paternity assignment of resulting offspring (Bangham et al. 2002). *st*^*1*^ had previously been serially backcrossed into the Dahomey genetic background (Bangham et al. 2002). To easily differentiate between males and females in mating treatments, *white* Dahomey females (w^Dah^) (Broughton et al. 2005) were used to permit focal males ad libitum mating opportunities. All genotypes were maintained prior to experiments at 25C, on SY media (10% m/v yeast, 10% m/v sucrose, 2% m/v agar, 0.3% v/v propionic acid, and 0.3% m/v nipagin) in a mix of bottles and large population cages, to maintain genetic diversity. For growing experimental flies, we used the standard larval density method (Clancy and Kennington 2001). At eclosion, virgins were sorted under light CO2 anaesthesia (< 5 min; < 5L/min; Flowbuddy, SLS); experimental males were placed onto their experimental diet within 6 h of eclosion. Diets were either lithium-supplemented or control (SY). In line with Castillo-Quan et al. 2016 LiCl (Merck) was dissolved in ddH_2_O at 5M concentration before supplementation to fly media. Equivalent volume of ddH_2_O were supplemented to control media to compensate for dilution. A salt was not used as a control vector since lifespan in *Drosophila melanogaster* has been found invariant to even very high concentrations (50mM) of NaCl (Castillo-Quan et al. 2016).

### Lifespan experiments

**Fig. 1:**
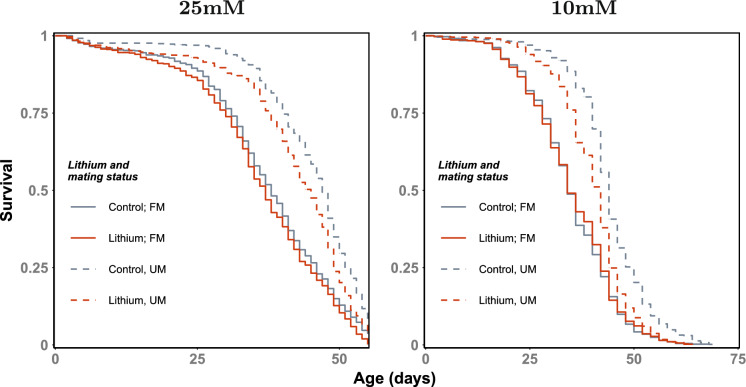
LiCl supplementation attenuates lifespan exclusively in unmated males. Two different experiments shown, with varying LiCl concentrations. 25mM experiment truncated due to experimenter availability. Note, different intervals in plots represent an experimental artefact of sorting (see M&M). *FM* frequently-mated, *UM* unmated. N = 2618 focal males for 25mM experiment; 2798 focal males for 10mM experiment. On average, n = 600–700 per group

Focal males were provided with ad libitum access to solid media, and maintained at 25C under a 12:12 light/dark cycle. Frequently mated males (FM) were maintained in at least a 2:1 female:male ratio, while unmated males (UM) were housed in single-sex groups. For FM treatments, females were replenished every 14 days to prevent co-ageing effects. Fly sorting was staggered over the course of two days for experiment 1 (Fig. 1 left-hand panel; 25mM LiCl) and in one day for experiment 2 (Fig. 1 left-hand panel; 10mM LiCl). Flies were scored for mortality and provided fresh media every other day. Given this, intervals in survival curves for experiment 2 are twice as large as those in experiment 1. Flies were housed in purpose-built demography cages (McCracken et al. 2020). UM cages contained 120 focal males per cage. FM cages contained 40 focal males and 80 females (maintained at ≥ 2:1 female:male), yielding an initial density of 120 flies per cage across treatments (n = 2600–2800 focal males per experiment). Both available vials were allocated the same experimental diet containing 4ml media. Escapees and extrinsic deaths (e.g. individuals lost during handling) were identified during scoring, facilitated by the distinct eye colours of focal males (red-eyed) and females (white-eyed), allowing individuals to be right-censored at the time of loss. Males used in reproductive assays were additionally right-censored, since they did not return to the lifespan experiment.

### Male reproduction assays

Reproductive assays were conducted at 5 weeks of age to quantify late-life reproductive performance following lifelong exposure, targeting the age at which diet-dependent reproductive ageing phenotypes are most strongly expressed. This timing was chosen because diet-mediated effects on male reproductive performance are often minimal at young ages and emerge more strongly later in life. Focal males at 5-weeks old were removed from experimental cages under light anaesthesia. Three days later we conducted mating trials. After removal from the cages, males were communally-housed in vials (n = 10) on their experimental diet. Virgin *st*^*1*^ females were sorted concurrently, and maintained in groups of 10. Therefore, females were 3-days old when housed with focal males. Females were provided with supplemental live yeast throughout, unless specified otherwise. Individual males and females were transferred into fresh SY vials on the day of the experiment, where focal male latency to mate was recorded (n ≈ 100 focal males per mating status and treatment). Females were then transferred to a new SY vial, and maintained for 48 h, prior to the remating experiment (where females were 5-days old); these vials were used to record offspring counts and determine male sterility. While offspring counts were carried out, these data were discarded due to adhesion of flies to media, once frozen. Males were considered sterile if there was no larval development from the vials in which their mates were housed after mating. Females who also produced no offspring after a remate were considered infertile and excluded from analyses. 48h following their first mating, females were then transferred into a new vial containing a 5-day old *st*^*1*^ male, where female remating latency was recorded. Females were then transferred into a fresh SY vial for 3 days, to assign paternity to any resulting offspring. For focal mating and remating experiments, a threshold of 4 min was used to exclude pseudomatings without sperm transfer. Experimental thresholds were set at 2 and 4 h, for focal mating and remating, respectively.

### Statistical analysis

All analysis was performed in R Statistical Software v4.1.2 (R Core Team 2020a). Data wrangling was performed using dplyr (Wickham et al. 2023) and tidyverse (Wickham et al. 2019), and figures were made using ggplot2 (Wickham 2016). Survival analyses were performed using survival (Therneau 2023) and coxme (Therneau 2022) packages. Generalised linear models (GLMs) were carried out using the stats (R Core Team 2020b) package. All models carried out were fully multiplicative ie included the interaction between LiCl supplementation and mating frequency. (Partial) likelihood ratio (LR) chi-squared tests served as our primary inferential control for family-wise Type I error when assessing whether a factor influenced the response (either alone or in interaction). When the LR chi-squared test indicated a significant effect for a factor (significant coefficient and/or interactive term containing a coefficient), we decomposed that effect by estimating marginal effects (conditional contrasts) of the factor at each combination of the other two binary variables. These marginal effects are reported with estimated differences and standard errors to show where the effect lies and its direction. Because the LR chi-squared test was the primary inferential test, these marginal effects are presented as descriptive, post-hoc decompositions to aid interpretation; the marginal-effect p-values are unadjusted and should not be treated as independent confirmatory tests. LR chi-squared testing of coefficients were performed using a ‘type II’ analysis of variance; ‘type II’ tests were chosen given their respect for the principle of marginality and inferential relevance (Langsrud 2003; Nelder and Lane 1995). Given this, all LR chi-squared tests reported in results all have 1 degree of freedom. Lifespan survival analysis was carried out using Cox mixed-effects models (Table S1, S3), using cage as a random term, with the exception of Table S2, where a Cox proportional hazards (Coxph) model was used. Table S2 serves as an illustration of the large between-cage variance inflating p-values in the Cox mixed-effects model. Coxph was also used for analysing latency and remating latency. The remainder of analyses for male reproduction were carried out using GLMs. Sterile mating analysis used a family of binomial (since dispersion parameter ≈ 1); p1 analysis used a quasibinomial family. Paternity share (p1) was calculated as the proportion of wild-type offspring among all offspring produced following female remating. Analyses of p1 were restricted to fertile focal males; this restriction did not alter qualitative conclusions.

## Results and discussion

We first tested the effect of lithium chloride (LiCl) on male lifespan using a concentration (25 mM) previously reported to extend longevity (Castillo-Quan et al. 2016). Both unmated (UM) and frequently-mated (FM) males were assayed to determine whether any effects of LiCl supplementation depended on the degree of reproductive investment. Across our dataset, we detected significant effects of both mating frequency (χ^2^ = 41.35, p < 0.001) and LiCl treatment (χ^2^ = 5.62, p = 0.018; Fig. 1; Tables S1–S2). However, when decomposing the LiCl effect to determine marginal effects (see M&M), we were unable to reject the null in a marginal effects analysis (Table S1), likely due to the truncation of the experiment and substantial between-cage variance in lifespan (see Table S2 for non mixed-model approach). The direction of the effect (HR ≈ 1.2) nonetheless suggested mild LiCl-induced toxicity.

To address this, we reduced the concentration of LiCl in the media to 10 mM—a dose also shown to improve lifespan in male *melanogaster* (Castillo-Quan et al. 2016)—and again assayed both lifespan and multiple metrics of reproductive success. At this lower dose, we once more observed a strong effect of mating frequency (χ^2^ = 72.35, p < 0.001), and the effect of lithium on lifespan now dependent on mating status (χ^2^ = 7.41, p = 0.0065; Fig. 1; Table S3). This interaction was driven exclusively by the response of UM males (HR = 1.53; SE = 0.14; p = 0.0023), indicating that lithium supplementation shortened lifespan in UM males but not in FM males.

To determine whether the detrimental effects of LiCl on lifespan were mirrored in late-life reproductive performance, we assessed pre- and post-copulatory reproductive traits in a subset of males drawn from the lifespan assay (Fig. S1). LiCl supplementation had no detectable effect on latency to mate (a measure of male attractiveness; χ^2^ = 0.11, p = 0.74; Fig. S1A), likelihood of sterility (χ^2^ < 0.001, p ≈ 1; Fig. S1B), or paternity proportion (χ^2^ = 1.09, p = 0.3; Fig. S1D), suggesting that lithium’s somatic effects can occur independently of reproductive performance. However, we did detect a significant interaction between LiCl and mating status on female remating latency—a proxy for a male’s ability to induce female refractoriness (χ^2^ = 8.62, p = 0.0033; Fig. S1C; Table. S4). This pattern was mostly driven by the reduced post-copulatory performance (higher female remating) of LiCl supplemented FM males (HR = 1.66; SE = 0.21; p = 0.018), demonstrating that the detrimental effects of 10 mM LiCl supplementation were not limited to UM males, but may also reduce quality of FM male ejaculate.

In this series of experiments, we examined the efficacy of a putative geroprotective compound in male *Drosophila melanogaster*, explicitly accounting for reproductive investment by comparing mated and unmated males across multiple dimensions of biological fitness. Unexpectedly, we found that the concentration of LiCl (25 mM) previously reported to extend lifespan instead reduced survival in our males. A lower concentration (10 mM) also impaired lifespan, particularly in UM males. Comparison of hazard ratios between experiments (25 mM HR = 1.245, CI [0.93–1.67]; 10 mM HR = 1.53, CI [1.16–2]) suggests that even lower concentrations are unlikely to produce neutral or beneficial effects.

In the study by Castillo-Quan et al. (2016), males were maintained in mixed-sex conditions for a brief period early in life before being separated by sex, representing an intermediate reproductive environment. In contrast, our design explicitly compared males maintained as virgins throughout life with those exposed to continuous mating. As such, our treatments do not directly replicate the reproductive conditions used in that study. Rather, our study was designed to test whether reproductive environment modulates the effects of lithium on survival. Under this framework, our results demonstrate that lithium’s effects are strongly dependent on mating environment, with no evidence of lifespan extension under either UM or FM conditions, suggesting that any putative beneficial effects are not robust across biologically-relevant reproductive environments.

While other methodological differences between our studies exist, these are unlikely to explain the contrasting effects of lithium on lifespan observed here. For example, Castillo-Quan et al. (2016) showed that high concentrations of NaCl (up to 50 mM) had no effect on lifespan in either males or females, indicating that the effects observed here are unlikely to arise from osmotic stress or altered feeding. Differences in housing conditions (vials versus demography cages) may also influence density, social interactions, and mating dynamics; however, such factors are unlikely to account for the consistent lifespan shortening observed here.

Having established that lithium does not extend lifespan under our experimental conditions, we next examined its effects on late-life reproductive performance. Males were largely unaffected by LiCl supplementation, with the exception of a decline in one aspect of post-copulatory performance among FM males. One possible explanation for this pattern is survivorship bias, given that only longer-lived individuals contribute to these assays. However, we consider this unlikely to explain our results. We detected no effect of LiCl supplementation on lifespan in FM males at 10 mM—the condition under which reproductive assays were performed—indicating that differential survival cannot explain the absence of LiCl effects on reproductive performance in this group. Accordingly, these findings point to an important, if tentative, conclusion: the effect of LiCl on lifespan is not necessarily coupled to any effects on reproduction.

Together, our results indicate that lithium’s biological activity in males operates within a mating-dependent context. Shorter-lived FM males were seemingly protected from the lifespan-reducing effects observed in UM males, suggesting that reproductive activity alters the physiological response to lithium. This could reflect a rightward shift in the reaction norm to LiCl among FM males—potentially due to lithium dissemination into the ejaculate, altered resource allocation under repeated mating, or overlapping metabolic costs between reproduction and lithium exposure.

Our results indicate that lithium’s effects on survival are strongly influenced by reproductive environment. While previous work has reported lifespan extension under specific conditions, our findings highlight that such effects may not generalise across different reproductive contexts. More broadly, these results emphasise the importance of considering environmental and life-history context when evaluating lifespan-modulating interventions.

## Supplementary Information

Below is the link to the electronic supplementary material.

Supplementary file1 (DOCX 15 KB)

Supplementary file2 (CSV 29 KB)

Supplementary file3 (CSV 28 KB)

Supplementary file4 (CSV 33 KB)

Supplementary file5 (PDF 290 KB)

## Data Availability

All data generated and reported in the manuscript is accessible as electronic supplementary material.
