# Supplementary material for: No evidence lithium supplementation extends lifespan in male Drosophila melanogaster
Source: Biogerontology. 2026 Mar 10;27(2):70. doi: 10.1007/s10522-026-10412-5 (PMC12975809; doi:10.1007/s10522-026-10412-5)
Supplement: Supplementary file 1 — Supplementary file1 (DOCX 15 KB) [file 10522_2026_10412_MOESM1_ESM.docx]

**Fig. S1. Male reproductive success at 5 weeks is largely refractory to 10mM LiCl supplementation. A:** Focal male latency to mate. **B:** Proportion of sterile focal males. **C:** Female remating latency. **D:** Focal male paternity proportion (p1). Estimates of **B** and **D** represented by means ± standard errors. FM = frequently-mated; UM = unmated. N = 357 focal males in **A**; 304 focal males in **B**; 298 focal males in **C**; 172 focal males in **D**.

Tables

**Table S1**. Marginal effects of 25mM lithium supplementation on male lifespan. Cox mixed-effects model.

| **TREATMENT** | **COEFFICIENT** | **HAZARD RATIO** | **SE (COEFF)** | **P** |
| --- | --- | --- | --- | --- |
| FM (NS) | 0.185 | 1.203 | 0.098 | 0.059 |
| UM (NS) | 0.219 | 1.245 | 0.15 | 0.15 |

**Table S2**. Marginal effects of 25mM lithium supplementation on male lifespan. Cox proportional hazards model.

| **TREATMENT** | **COEFFICIENT** | **HAZARD RATIO** | **SE (COEFF)** | **P** |
| --- | --- | --- | --- | --- |
| FM | 0.179 | 1.2 | 0.056 | 0.0015 |
| UM | 0.247 | 1.28 | 0.06 | <0.0001 |

**Table S3**. Marginal effects of 10mM lithium supplementation on male lifespan. Cox mixed-effects model.

| **TREATMENT** | **COEFFICIENT** | **HAZARD RATIO** | **SE (COEFF)** | **P** |
| --- | --- | --- | --- | --- |
| FM (NS) | -0.034 | 0.967 | 0.0941 | 0.72 |
| UM | 0.423 | 1.526 | 0.139 | 0.0023 |

**Table S4**. Marginal effects of 10mM lithium supplementation on female remating latency. Cox proportional hazards model.

| **TREATMENT** | **COEFFICIENT** | **HAZARD RATIO** | **SE (COEFF)** | **P** |
| --- | --- | --- | --- | --- |
| FM | 0.505 | 1.657 | 0.214 | 0.018 |
| UM (NS) | -0.354 | 0.702 | 0.202 | 0.08 |
