# Supplementary figures and images for: No evidence lithium supplementation extends lifespan in male Drosophila melanogaster
Source: Biogerontology. 2026 Mar 10;27(2):70. doi: 10.1007/s10522-026-10412-5 (PMC12975809; doi:10.1007/s10522-026-10412-5)

**A**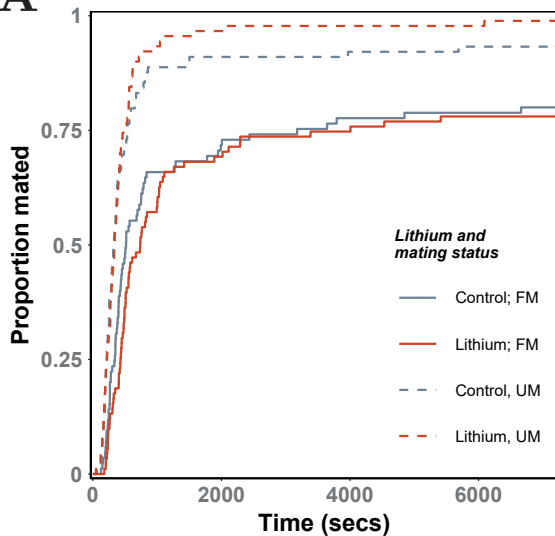**B**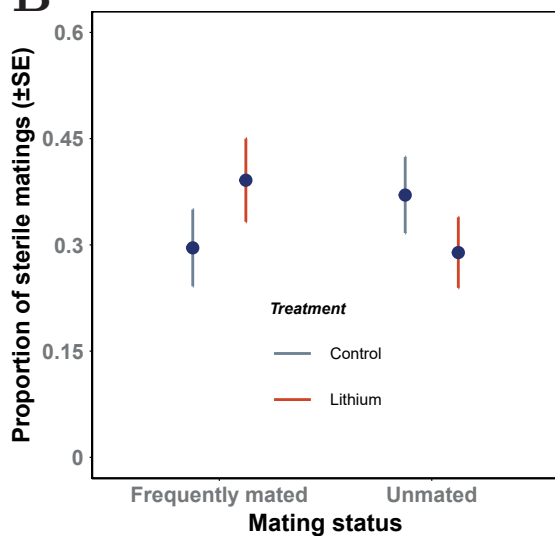**C**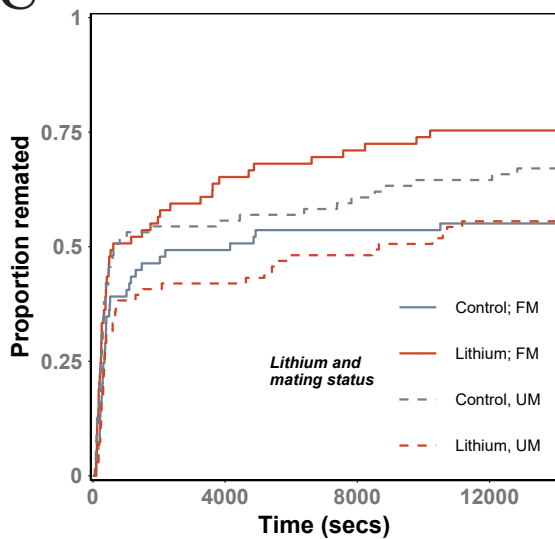**D**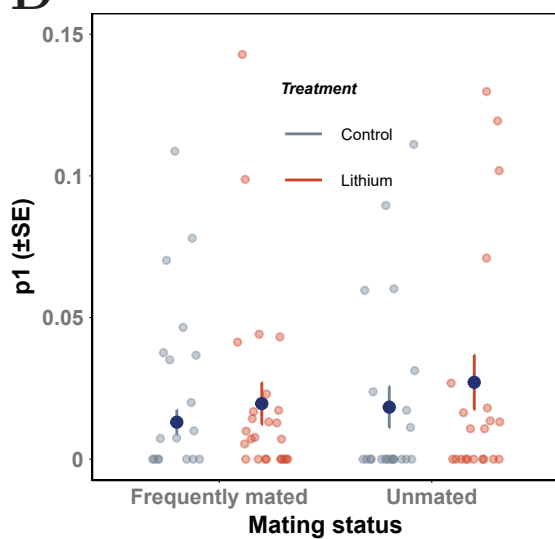

Supplement: Supplementary file 5 — Supplementary file5 (PDF 290 KB) [file 10522_2026_10412_MOESM5_ESM.pdf]
